# *You Are Not Alone*: The (In)Visible Homeless and the Role of Social Workers and Related Professionals

**DOI:** 10.3390/ijerph191610070

**Published:** 2022-08-15

**Authors:** Ariadna Munté-Pascual, Gisela Redondo-Sama, Irene De Vicente, Virginia Matulic

**Affiliations:** 1 Social Work Training and Research Section, Faculty of Education, University of Barcelona, 08035 Barcelona, Spain; 2 Department of Pedagogy, University Rovira i Virgili, 43007 Tarragona, Spain

**Keywords:** social work, homeless people, health information, pandemic

## Abstract

The scientific literature has already shown that health information is a factor that contributes to reduce health disparities, improving the situation of vulnerable groups, such as homeless people. However, less is known about the ways that health information has been spread by social workers and related professionals to homeless people in the first moments of the COVID-19 pandemic. This work analyses some social work actions related to health information addressed to homeless people and to identify its impact during the lockdown in Barcelona. This qualitative study is based on semi-structured interviews with social workers and related professionals involved in developing interventions related to health information with homeless people in Barcelona. The data analysis is based on the communicative methodology to identify exclusionary and transformative dimensions. The results show the improvement in the health information of homeless people during the pandemic through the design and development of actions facilitated and promoted by some social workers and related professionals. The findings demonstrate the role that some social workers and related professionals have played in the improvement of health information of homeless people during the lockdown in Barcelona.

## 1. Introduction

The scientific literature shows that health information is one of the factors that improve people’s health and prevent diseases, with an increased interest by the scientific community in analysing the access and barriers to online health information [1]. In this vein, health literacy programmes have also promoted the access and use of health information to reduce health disparities, particularly in vulnerable groups [2], around the globe. In this section, we explain some of the recent contributions in the field of health information in the COVID-19 context, the role of social work, and the reality of homeless people.

Several recent studies place the relevance of approaching the situation of homeless people in the pandemic context [3,4,5,6]. This reality has been analysed in diverse countries and regions worldwide, advancing knowledge about different issues affecting this vulnerable group, some of them contributing to provide solutions. For instance, in the European context, Roeder et al. [7] describe the seroprevalence and risk factors of exposure to COVID-19 in homeless people in Paris (France) through a cross-sectional study at food distribution sites, emergency shelters, and workers’ residences attended by medical services. The results show that between 23 June and 2 July 2020, 52% of recruited people tested positive, with a high exposure to SARS-CoV-2 and with relevant variations at different study sites (workers’ residences, emergency shelters, and food distribution sites). The study highlights the importance of providing safe and uncrowded accommodation, including the need for adequate testing and public health information. In the case of Germany, Graske, Koppe, Neumann, and Forbig [8] also developed a cross-sectional study in five German cities to identify the limitations of services for homeless people during the COVID-19 pandemic. The authors identified that the institutions reduced their operating hours and capacity for guests and reported negative impacts regarding the anxiety and desperation of homeless people, among other aspects. This type of descriptive work with evidence of the situation of homeless people allows for the design of preventive measures and the ways social workers and related professionals, health professionals, and general services for vulnerable groups can foresee and act better against risk factors.

The responses of public services to the needs of homeless people during the pandemic are investigated in different countries, for example, by Schiff, Krysowaty, Hay, and Wilkinson in Northwestern Ontario in Canada [9]. The authors analyse the pandemic preparedness and response in service hub cities, going in-depth into a case study focused on a small city that experienced a serious outbreak of COVID-19 amongst homeless persons and shelter staff in the community. This study provides evidence about the need to increase support to the homeless sector in small service-hub cities, create pandemic plans, and end chronic homelessness in some deprived regions, such as rural areas. The lessons learned can be potentially useful for other cities nationally and internationally of similar size, geography, and socio-economic position, advancing knowledge towards the replicability of scientific results. Furthermore, it is important to highlight that the authors mention the situation of the Indigenous population in these territories, paying special attention to diverse vulnerabilities that influence disadvantages. Still in the Canadian context and specifically analysing the role of social workers in Nova Scotia, Wu and Karabanow [10] provide recommendations about the social work strategies to reduce homeless people´s vulnerabilities and provide the safe communities they deserve.

The young people experiencing homelessness in the United States are at the core of the work of Tucker et al. [11], which aimed to address how the COVID-19 outbreak has affected emerging homeless adults concerning engagement in protective behaviours, mental health, substance abuse, and access to services. Participants were enrolled in a substance use and sexual risk reduction intervention for 18 to 25 years old experiencing homelessness, and most of them reported engaging in COVID-19-protective behaviours. Particularly, the questions administered between April and July 2020 to participants were addressed to learn about COVID-19 in four aspects: knowledge and perceived susceptibility, protective strategies, mental health and substance use, and the ability to address needs. With regards to health information, the participants were asked about where they obtain information about COVID-19, with results showing that 71% were from social media, 59% from TV/radio, 60% from news websites, 52% from friends or family, 45% from service providers or health professionals, and 42% from newspapers. Moreover, the results show an increase in mental health symptoms from the COVID-19 outbreak, such as hopelessness, anxiety, or depression, and an increase in alcohol, tobacco, or marijuana use among those previously using substances. In the light of the findings, the authors argue the need for innovative strategies to respond to the increased service needs of young adults homeless during outbreaks.

The analysis of homeless people in the COVID-19 pandemic was also developed in the Asian context. In the case of Japan, Fujii [12] develops a study to analyse the demographic characteristics of homeless people, as well as their anxieties and if the weekly announcements about COVID-19 were beneficial for them. Concerning health information, the author concludes that 78.6% of the respondents found the health announcements to be helpful and took preventive measures. Moreover, it is also argued that there is a need to continuously provide support for Japan´s homeless population. Moving from Japan to India, Gowda, Chithra, Moirangthem, Kumar, and Math [13] approach the impact of lockdown policy on homeless people, in particular homeless persons with mental illness, paying special attention to the efforts to provide care and protection.

The need to provide health information to homeless people in the COVID-19 pandemic is central in some works developed from diverse approaches and perspectives. Tagliacozzo, Albrecht, and Ganapati [14] departs from the communication ecology to examine the online communication of national public health agencies during the pandemic in three countries: Italy, Sweden, and the United States. The work focuses on the coordination of online communication of national public health agencies with other agencies and organizations and the ways online communication from other agencies was diversified for targeting specific organizations and social groups. The authors developed a content analysis of Twitter data and agency press releases showing differences in each country but with a common a lack of tailored information from the agencies to several vulnerable groups, including homeless populations, for which no messages were addressed. This absence has negative consequences for vulnerable groups, which requires actions to adapt communication strategies and efforts to reverse this situation. Moreover, some works focus in one specific country, as in the case of France approached by Crouzet et al. [15]. The authors identify the experiences and perceptions of the vulnerable population, specifically homeless people and migrants, through qualitative data to understand how they perceive and react to the measures to limit COVID-19 infections. The content analysis of the interviews implemented in seven shelters in Lyon and Paris shows a reminiscence of prior violent or traumatic circumstances for the participants, conflicting visions of the shelters, an abundant flow of information with impact on participant´s wellbeing, and limitations to daily life activities. Concerning information, the authors conclude that it is crucial to improve not only its availability but also health literacy.

Within this framework, and as a justification for this study, the research addresses the need to investigate the role that some social workers and related professionals have played during the lockdown in Barcelona to provide health information to homeless people. Barcelona is a city located in the northeast of Spain with a total population of 1,636,732 [16], and according to data from 2021, it has 4845 homelessness people [17] with the following profiles: 91% are men, the median age is 43, 68% come from other countries, the average time spent living in the street is 4 years, 59% declare poor health, and 46% have received physical and/or verbal aggressions. It is important to highlight that the strategy to obtain the data is obtained from a re-count conducted during one night, when a team of professionals and more than 500 volunteers went out to the streets to count and interview people. At the national level, the official data from the National Institute of Statistics shows in 2020 a daily average of 17,772 homeless peoples housed in shelters and other types of State accommodation centres, with an increase in women attending [18]. However, there is a lack of national data regarding the total number of homeless people living in the country. Within this framework, the role of social work and related areas becomes crucial. There is evidence of programmes related to social work implemented in the mental health field [19] in the pandemic context, and this work aims to advance this knowledge to show the relevance of social work in such a challenging context.

## 2. Materials and Methods

### 2.1. The Study

The state of alarm and the Spanish lockdown was declared by the government on 14 March 2020, with limits to national mobility and defined containment measures. In this context, there were some basic services not suspended because of their crucial role to keep the population safe, as in the case of social workers and related professionals and health professionals, among others. This situation ended on 21 June 2020, although the measures to prevent the spread of COVID-19 have continued to the present. In this period of time, and particularly at the beginning of the national lockdown, social workers and related professionals were at the front line facing some of the most challenging situations for vulnerable groups such as homeless people.

### 2.2. Methodology Design and Procedure

This work is based on the communicative methodology (CM) [20], which integrates the exclusionary and transformative dimensions in the research process. In this vein, the CM allows the identification of actions that contribute to overcoming disadvantages and exclusion, through an egalitarian dialogue between the research team and the people being interviewed. Furthermore, it is a methodology used in qualitative research with homeless people [21]. Through a qualitative design, the implementation and analysis of semi-structured interviews to social workers and related professionals, the objective of this work was to identify their experiences and roles concerning the health information provided to homeless people. The fieldwork was performed during the first 15 days of the lockdown in Barcelona, one of the most affected areas worldwide at the beginning of the pandemic.

The International Day of Social Work is celebrated on 17 March, and in 2020, the research team decided to create a WhatsApp group to share potential ideas to collect evidence of what was happening in the daily life of social workers and related professionals working with homeless people, among others, in such a challenging context. One of the ideas was to initiate contacts with social workers and related professionals that would like to be part of the research process by explaining their feelings and experiences. Then, the research team used different communication channels (email, telephone, or WhatsApp) to contact social workers and related professionals with whom there were previous collaborations to propose the participation in the study. Most of them reacted very positively, and the research team facilitated the possibilities to concretise how to develop the semi-structured interviews. Once the initial contacts were ensured, the team selected the settings and participants in the field of homeless people.

The semi-structured interviews were developed in a very challenging context, as the professionals were facing a totally new and unpredicted situation. Therefore, the research team designed very diverse strategies to reach and collect the information, using different communication channels (telephone, video calls, etc.). The priority was to not disturb the work the social workers and related professionals were doing, but to capture the essence of their tasks. The questions underlying the semi-structured interviews included the following topics:

- Experience of the professional interviewed;
- Impact on the personal life of the professional;
- Experience of the professional team;
- Experience of the attended people;
- Difficulties found;
- Organizational responses of the support and protective measures;
- Teamwork and networks among professionals;
- Limitations in the provided support;
- Challenges in emergency situations;
- Solidarity;
- Other aspects.

### 2.3. Type of Organizations and Participants

The entities and programmes working in the field of homeless people that were included in this study have similar aims with slight divergences, which are summarized in what follows.

Entity-programme 1. This entity was founded in the 1980s with the objective to attend to and advise homeless people living on the streets. Professionals working in this entity accompany homeless people to achieve the best possible autonomous life, covering their basic needs, providing social and health support, and ensuring accommodation. Furthermore, they also work to raise public awareness, denounce unfair situations, and provide solutions to administrations and civil society to make sure nobody sleeps on the streets. In the first moments of the COVID-19 outbreak and lockdown, the entity activated a new team for the streets and a telephone service to face the lockdown situation of homeless people.

Entity-programme 2. This entity was created in the 2010s with the aim of empowering women to be able to reverse their homelessness through projects that guarantee a feminist approach and community action. The entity provided safe spaces, individualized work plans and community work as a tool for collective empowerment. They manage projects for homeless women offering accommodation, food, and transport assistance, as well as social support to help them start an independent life. During the first moments of the situation of COVID-19, they were forced to close their premises, and the monitoring of women was developed through teleworking and coordination with other services.

Entity-programme 3. This is a program promoted by the entity-programme 1 to respond specifically to the lack of medium- and long-term residential resources for homeless people who are affected physically and mentally. Their care is socio-sanitary, due to the fact that the attended people have significant health difficulties that require intense and continuous care. Due to the situation of COVID-19, professionals from other resources joined this programme, thus protecting some of the professionals (teleworking) from possible work leaves due to infection. The entrances and exits were restricted.

For this study, the selected social workers and related professionals came from these three entities-programmes, with different trajectories and years of experience working with homeless people, as shown in Table 1 and Table 2. During the first 15 days of lockdown in Barcelona, the research team started to contact social workers and related professionals from diverse fields of intervention. The total number of social workers and related professionals initially included was 28, and most of them expressed their willingness to participate in the study despite the challenging situation. Finally, there were seven participants working in the field of homeless people, including one participant from the field of homeless women with the following experiences and duties:

- Juan. He belongs to a working group dedicated to attending to people in the street, with direct and personalized interventions. In the first days of the lockdown, his task was to visit people sleeping in the streets to accompany them and check their health status and provide solutions to the emerging problems.
- Carmen. She belongs to a working group dedicated to attending homelessness people.
- Jessica. She provides direct and group intervention to homeless women.
- Santiago. He provides direct and group intervention to homeless people.
- María. She provides direct and group intervention to homeless people.
- Sonia. She provides direct and group intervention to homeless people.
- Ana. She provides direct support to homeless people, in particular by providing information, promoting feelings of belonging, and accompaniment.

### 2.4. Data Collection and Analysis

The semi-structured interviews were developed in March to April 2020, in the worst moments of the pandemic in Spain. Taking into account the difficulties of developing the fieldwork face-to-face due to the lockdown, the research team offered different options to the social workers and related professionals using online possibilities, such as email, telephone, and WhatsApp. Moreover, the social workers and related professionals decided when was the best moment to conduct the interview, and flexibility was ensured in the whole research process so as to not disturb their emergency work. The data collection featured a dialogic process in which the concerns of social workers and related professionals and how they were facing the situation were raised. According to the selected methodology, the guiding questions included the transformative dimension of the actions undertaken by the social workers and related professionals.

Each participant provided general information about their individual background, including their years of experience in social work and the specific tasks developed with homeless people. The research team systematized the data recording the conversations and taking notes, transcribing and selecting the relevant quotations about health information for homeless people. There was a template to transfer and share the data, including the categories emerging from the semi-structured interviews with social workers and related professionals.

### 2.5. Ethics Statement

This work was based on the information provided to the participants about the purpose of the study, the voluntary bases, the permission to publish the data, and the anonymity as all the names are not real. Informed consent was obtained and dully saved, providing the participants with a certificate.

## 3. Results

### 3.1. Lack of Information, Over-Information, and Misinformation

The data analysis shows that homeless people did not receive effective and clear information about the health emergency, as well as the measures that the majority of the population had. Furthermore, this situation was generated not only by the immediate context of homeless people from the entities that give them support but by other agents of the community, such as the police or neighbours. Furthermore, it was a fact that the over-information contributed to generating continuous changes in the steps to take, contributing to creating confusion among homeless people and social workers and the related professionals attending them. Juan highlights these aspects as follows:

“We could see that many [homeless people] were disconcerted, they received contradictory information from the neighbours and other social agents, mainly the police, and this generated concerns for them (…) This would be a third difficulty: the over-information we receive and the people we care for. We are all very nervous. I think it’s something that’s happening all over society, we’re all more connected than ever, and people are constantly sending new messages and information. At the social sector level, this is also happening. From the day the lockdown began, we have received news about new resources that would open and others that would close and this information was changing every day. I think the administration has not been at all clear in this regard, they have announced many things, but then they were nuanced and this has created a state of confusion among people sleeping on the street, among professionals, and also among other social agents (neighbours, police, …) who interact with people sleeping on the street”.

Similarly, Jessica mentions that the lack of information created difficulties in developing their tasks as social workers and related professionals and the feeling of not knowing the impact of their tasks:

“The response was slow and there was a lack of information. We don’t know how it’s working and what we’re doing. The lack of information greatly disrupted the work”.

Within this context of a lack of information, the very basic needs were not properly covered, for instance, a place to shower or eat, as mentioned by Juan:

“Many social resources for the homeless people have closed or limited their hours and this has left them even more helpless and having difficulty accessing such basic things as a place to take a shower or eat (…) These days, with empty streets, we see a lot of people sitting on a bench or lying in a garden, and with the excuse of asking them how they are in terms of health, we are meeting homeless people we didn’t know until now. The invisible ones have suddenly become more visible than ever”.

The use of sources of information was highlighted by Ana to explain that the homeless people did not have the opportunity to be informed by themselves. According to this, improvement is needed in communication channels specifically designed for and addressed to vulnerable people:

“Attended people have no resources to look for and misinformation in this regard is something that is clear these days. These days I realize that information and communication is influenced by social classes and people on the street are the first to receive this impact and stay out of communication. (…) Disinformation is an important point in this health emergency situation, and I believe that clear and concise communication channels should be developed for the population in a situation of social exclusion, which can reach everyone and people can understand the severity of this situation and the need to protect oneself, to be alert to symptoms and how to proceed if they appear”.

### 3.2. Concrete Barriers and Measures While Working with Homeless People

Some social workers and related professionals describe the perceptions and feelings that homeless people may have, and the barriers they face to follow the explanations regarding, for example, social distancing. Homeless people used to have a strong relationship with social workers and related professionals, and sometimes they may act as the main channel of communication in their daily lives. Because of this, the social workers and related professionals realized how important it was to listen and take care of them to try to raise awareness of the difficulties faced, as Juan describes:

“We have to think that they are people with a lot of difficulties and that they are in very complex situations. They are not always able to follow the directions we give them, they are very close to us and it is difficult for them to understand that they have to keep their distance. At other times we are the ones who have to approach them, because they feel bad or because they speak very softly and we don’t listen to each other”.

The lack of information was progressively improved, and the measures to prevent infection became more accepted by homeless people. This was possible because of the social workers and related professionals contributing to disseminate the health information considering the different stages and processes of the attended people. Santiago describes in an accurate way the different moments of this process, also mentioning the ways that homeless people were reacting. In this vein, it is important to mention that each person had the capacity to decide what to do but with knowledge about the situation and measures. Furthermore, this social worker identifies the sharing of data between homeless people, staff, and other users, an aspect that improved their work, as well as a concrete example of the hygiene standards they facilitated:

“In the case of the attended people (living in residential care, but who have previously lived, in some cases, for long periods on the street) and according to my interpretation, I think there have been various stages they have gone through initial disbelief and rejection of reality. Then, it came to the anger expressed against us for being the ones who were carrying out the surveillance that the confinement measures were maintained and the ones who had to take drastic measures to prevent the exit and free access, to which they use to do… In most of them, the information has been internalized and they have joined in protecting and maintaining the precautionary and hygiene measures, which the staff has also been constantly reminding them. Finally, each one of them is facing the situation in their way, some with resignation, others inventing excuses to skip the confinement, others informing themselves and sharing the data with the staff and with other users, others scared, and others aware of the cause (…). The protective measures that we are following in the service are those indicated by the Ministry of Health, distancing between people, use of masks and gloves in the moments of greatest proximity to the person served (hygiene, showers, postural changes, delivery of medication, food, etc.). We have favored and promoted hand hygiene standards among our users”.

Another social worker, María, explains the limits of their tasks and many professionals working with homeless people were performing telework, mainly to avoid the risk of infection. She describes the organization of the entity-programme to provide support in concrete aspects, such as the hygiene guidelines:

“As in this entity-programme we have many professionals doing teleworking, what we have done is take advantage of them and that they can come and help us. We need a lot of help with hygiene guidelines and residents’ standards”.

The health information included the use of information posters, clean hands, and safe distances between people, as well as wearing masks, as explained by Sonia:

“To raise social awareness of the situation of people living on the streets who are currently unprotected as many resources have closed and there are many difficulties in covering food, hygiene and rest for people. The main protection measures include: placing information posters, mention the importance of clean hands and safe distance between people living there and wear masks and gloves at all times”.

## 4. Discussion

The social workers and related professionals participating in this study have contributed to understanding the reality of homeless people and health information during the beginning of the lockdown in Spain, which is a research topic underexplored in the scientific literature. Therefore, the results allow the advancement of knowledge complementing studies implemented in other European and non-European countries, such as France [7] or Germany [8] and Canada [9,10] or Japan [11]. Their reflections, feelings, and experiences focus on diverse aspects of information, from the lack of information to over-information and misinformation. In this vein, previous studies have raised concerns about the limitations and barriers to access health information in vulnerable groups [6], an aspect aggravated in the COVID-19 pandemic context in spite of the importance of approaching the situation of this vulnerable group [3,4,5,6].

It is also important to mention that the professionals interviewed have shared concrete situations of homeless people and the ways they react to the health information provided, in particular the protective measures. This aspect related to the protective measures has not been specifically identified in the scientific literature, which enriches the analysis of homeless people during the lockdown.

Overall, the participants of the study are consistent with the description of the situation of homeless people, raising concerns about how to overcome the barriers regarding information and putting providing support to them as a priority. This result is aligned with contributions in the field, including online communication [5,12,14].

There is evidence of how health information influences health status, increasing the opportunities to live in better conditions [2]. However, we have identified in the literature review that there is a gap in analysing particularly how health information from some social workers and related professionals has been used to alleviate the pandemic and lockdown consequences. As identified in the introduction, there are descriptive works and contributions in the field but with a scarcity of focus on social work and related professionals in spite of some efforts [11,19], which has been crucial to attending to vulnerable groups. The health professionals have been at the frontline of COVID-19, but the vision of who works on the frontline [22] needs to be expanded to other professionals that have been in a similar position, being at risk of or becoming infected because of their work.

Therefore, it is necessary to go in depth into the experiences of social workers and related professionals in their aim to overcome the social exclusion of homeless people, paying special attention to their views regarding the management of health information. This will allow us to increase their potential benefit to vulnerable groups, in collaboration with other agents, both public and private ones.

The future research in this field can illustrate emerging forms and effective strategies to support the role of social workers and related professionals to prevent emergency situations. This work provides evidence about the need to further develop effective channels of health communication with homeless people, identifying emerging successful strategies that require more investigation. Considering that the period of this study focuses on the first 15 days of the lockdown in Barcelona, it inspires future works to provide supporting evidence through better channels of communication. This will contribute to minimizing the risks of increasing the vulnerability and exclusion of those who are on the margins of societies.

## 5. Conclusions

The inclusion of social workers and related professionals in research is needed to better understand the situation of homeless people during COVID-19 in order to provide evidence-based solutions related to health information to improve the work of these professionals. Therefore, it is important to expand the bottom-up strategies that enable entities and programmes to raise awareness of the situation of vulnerable groups. Furthermore, it will be crucial in future works to analyse the synergies between the situation affecting homeless people and the decisions made by institutions, also influenced by the limited information about COVID-19 and its impact on vulnerable groups. While most of the citizens were in their houses during lockdown, the homeless people were more visible than ever, making the work of those dedicated to improving their lives more necessary.

## Figures and Tables

**Table 1:** Participants’ profiles.

ID	Gender	Field of Intervention	Age Working in the Service	Age
Juan	Male	Homeless	8	30
Carmen	Female	Homeless	3	29
Jessica	Female	Homeless women	15	44
Santiago	Male	Homeless	6	32
María	Female	Homeless	10	35
Sonia	Female	Homeless	6	31
Ana	Female	Homeless	8	28

**Table 2:** Mean age in service and age.

Mean Age Working in the Service	Mean Age
8	32

## Data Availability

The interviews were recorded. They are anonymized and safety stored. Only the authors who conducted the interviews have access to this material in order to protect the confidentiality and privacy of the participants.
